# Case Report: Co-infection with SARS-CoV-2 and influenza H1N1 in a patient with acute respiratory distress syndrome and a pulmonary sarcoidosis

**DOI:** 10.12688/f1000research.26924.2

**Published:** 2022-05-03

**Authors:** Lekbir Baala, Dalila Benzekri-Lefevre, Laurent Bret, Toufik Kamel, Clémence Guillaume, Laura Courtellemont, Abdelkrim El Khalil, Thomas Guery, Sophie Iquel, Olivier Perche, Khalid Khadre, Thomas Brungs, Julien Decker, Thomas Francia, Julie Bois, Benoit Delamare, Jérôme Guinard, Laurence Got, Sylvain Briault, Thierry Boulain, Eric Legac

**Affiliations:** 1 Pole de Biopathologie , CS 86709, 45067 Orléans CEDEX , France, Centre Hospitalier Régional d’Orléans, 14 Avenu de l’Hôpital, Orléans, France; 2 UMR7355 INEM Immunologie et Neurogénétique Expérimentales & Moléculaires, CNRS & Université d'Orléans, 3B rue de la Ferollerie, Orléans CEDEX 2, 45071, France; 3 Service de Médecine Intensive Réanimation, Pole Métiers de l’Urgence, Centre Hospitalier Régional d’Orléans, 14 Avenu de l’Hôpital, CS 86709, Orélans, 45067, France; 4 Service de Pneumologie, Centre Hospitalier Régional d’Orléans, Orléans, 45067, France; 5 Service de Radiologie, Centre Hospitalier Régional d’Orléans, Orléans, 45067, France

**Keywords:** Co-infection, SARS-CoV-2, Influenza H1N1, Pulmonary sarcoidosis, Comorbidity

## Abstract

Coronavirus disease 2019 (COVID-19) is caused by severe acute respiratory syndrome coronavirus 2 (SARS-CoV-2) infection and has been a global public health concern. We report coinfection of SARS-CoV-2 and 2009 H1N1 Influenza strain in a French patient with pneumonia leading to acute respiratory distress syndrome.  The patient also had a medical history of pulmonary sarcoidosis with a restrictive ventilatory syndrome and obesity, which would be a supplementary risk to develop a poor outcomes. This case highlights the possible coinfection of two severe SARS-CoV-2 and influenza H1N1 viruses in comorbid patient, which presents a higher risk to extend the care duration. The overlapping clinical features of the two respiratory syndromes is a challenge, and awareness is required to recommend an early differential diagnosis and it’s necessary to adopt the vigilant preventive measures and therapeutic strategies to prevent a deleterious impacts in patients with comorbid factors.

## Introduction

Coinfections involving SARS-CoV-2 and respiratory viruses influenza viruses (A or B) have been rarely reported ^1– 9^. One recent meta-analysis indicated that overall 1.2% of COVID-19 patients had influenza co-infection ^10^. However, there was no more evaluation of the infection effect in clinical outcomes and less in patients with other comorbidity factors. Our patient presented concurrent COVID-19 and Influenza infections with a history of pulmonary sarcoidosis.

## Case report

A 41-year-old man presented to the hospital’s emergency unit with fever and cough that has been progressing for several days. The SARS-CoV-2 RT-PCR test carried on the 3 targets genes *RdRp, N, E* was positive on March 22 ^nd^ 2020, wheares the cough and fever had already been evolving for three to four days before. On March 24 ^th^ 2020, the patient had developed a dyspnea aggravation and was taken care of by a medical unity at home. He has a medical history of pulmonary sarcoidosis with a restrictive ventilatory syndrome, which was being treated with Methotrexate (15mg per week) and folinic acid (0,4mg one tablet per day). He also had malaria in 2004 from a trip to Central Africa.

Physical examination on the 24 ^th^ March revealed a respiratory rate of 41 breaths/minute (normal range (NR) 12–20 breaths/minute) and oxygen saturation SpO2 of 75% (reference range (R-R) 95–100%) on ambient air. The SpO2 became at 96% when given mask flow oxygen at a rate of 12l/minute. The patient was transferred to the emergency room with 97% SpO2, body temperature 37.2°C, and respiratory rate of 30 breaths per minute. The patient presented with superficial polypnea, dyspnea with little effort, difficulty in speaking and bilateral “crackles”. Neurological and cardiovascular examinations were normal.

On supplemental oxygen (12l/min), arterial blood gas analysis revealed pH 7.50 (R-R 7.35–7.45), PCO _2_ 35 mmHg (NR 35–45 mmHg), PO _2_ 88 mmHg (NR 75–100 mmHg), HCO3- 27.3 mmol/l (R-R 22–26 mmol/l), and SaO2 94.4% (R-R 95–100%). The patient was then transferred to intensive care unit (ICU). Respiratory panel tests were negative for adenovirus (subtypes 2, 3, 6, 7.1 and 8), coronaviruses (229E, HKU1, NL63 and OC43), human metapneumovirus, rhinovirus, enterovirus, MERS-CoV, parainfluenza virus (1,2,3 and 4), respiratory syncytial virus, and *Bordetella pertussis* and *parapertussis*. However, influenza A, subtype influenza A-H1 variant 2009, was positive. The molecular and kenetic analysis of the 3 target genes ( *RdRp, N, E*) of SARS-CoV-2 revealed the persistence of virus for 3 weeks.

A chest computed tomography scan revealed a predominant left interstitial lung condensation syndrome. Routine laboratory tests revealed higher parameters during patient hospitalization: creatine phosphokinase 2999 UI/l (R-R 30–200UI/l); gamma glutamyl transferase 119 Ui/l (R-R 12–64UI/l); D-Dimers, which has increased two fold in one week, 3620 ng/ml (April 6 ^th^) to 7520 (April 15 ^th ^) (R-R 40–500UI/l). Other parameters were also elevated: fibrinogen 8.58g/l (R-R 2–4g/l); aspartate-aminotransferase 55 UI/l (R-R 5–34UI/l); platelets 599x10e9/l (April 7 ^th^) (R-R 150–450x10e9/l); leukocytes 15.4x10e9/l (R-R 4–10x10e9/l) ( Table 1).

**Table 1.** Laboratory findings in the patient with coinfection of SARS-CoV-2 and influenza H1N1. NA: data non available.

Laboratory parameters (reference range)	March, 26	March, 26	March, 31	April, 03	April, 04	April, 07	April, 10	April, 11	April, 12	April, 13	April, 15	April, 17	April, 24	April, 30	May, 06
Leukocytes (4–10x10e9/l)	8.8	12.4	11.8	11	11.5	10.5	15.4	15.2	11.4	11	13.8	14.7	13.2	10.7	6.4
Red cells (4.50–6.50x10e12/l)	5.08	4.88	NA	NA	4.38	4.18	4.19	4.33	4.07	3.99	4.25	4.21	4.57	4.59	4.81
Hemoglobins (13–17 g/dl)	13.4	12.7	12.1	11.6	11.2	11	10.8	11.3	10.5	10.3	11	10.8	11.9	12.1	12.8
Hematocrit (40–54%)	40.7	38.9	36.5	36.4	35.2	33.3	33.9	34.9	33	32.3	34.1	33.7	36.6	37.2	39.4
Mean corpuscular volume (80 um3)	80	80	79	81	NA	80	81	81	81	81	80	80	80	81	82
Mean corpuscular haemoglobin concentration (27–32 pg)	26.5	26.1	26.2	25.7	25.6	26.4	25.7	26	25.8	25.9	25.1	25.8	25.9	26.3	26.6
Red cell distribution width) (12–16%)	15.9	NA	NA	NA	NA	NA	NA	16.2	16.5	16.2	15.4	15.6	16.8	17.9	18
Platelets (150–450x10e9/l)	126	NA	180		528	599	556	516	437	382	336	296	311	284	218
Total neutrophils (1.80–8x10e9/l)	7.98	11.23	10.83	8.69	8.75	6.52	12.26	12.04	8.66	8.82	12.1	11.32	8.96	7.6	4.19
Total lymphocytes (1–4x10e9/l)	0.68	0.73	0.5	0.88	1	1.79	1.56	1.66	1.23	1.31	1.02	1.78	2.56	2.1	1.31
Total monocytes (0.20–1x10e9/l)	0.13	0.36	0.41	0.88	1	1.23	1.36	1.11	1.16	1.08	0.64	1.19	1.16	0.81	0.65
D-Dimers (40–500 ng/ml)	NA	NA	NA	NA	NA	3620	3800	NA	4000	5660	7520	NA	NA	3070	NA
Fibrinogen (2–4 g/l)	NA	NA	NA	NA	NA	8.59	6.58	NA	6.23	5.68	6.12	NA	3.57	2.86	NA
Blood protein (64–83 g/l)	57	NA	53	56	55	61	NA	61	56	57	66	63	NA	68	68
Blood creatinine (64–104 µmol/l)	80	NA	NA	NA	NA	NA	NA	NA	60	NA	46	53	61	67	NA
Urea (3.2–7.4 µmol/l)	NA	NA	NA	9.9	10.3	11.1	NA	9.8	10.2	9.2	5.1	NA	NA	NA	NA
Creatine phosphokinase (30–200 UI/l)	2999	1473	NA	415	602	NA	NA	NA	111	NA	NA	NA	NA	34	NA
Aspartate aminotransferase (5–34 UI/l)	NA	55	NA	NA	45	NA	NA	NA	23	NA	NA	NA	13	NA	29
Gamma glutamyl transpeptidase (12–64 UI/l)	NA	119	NA	109	110	90	NA	81	66	NA	NA	58	51	NA	35
Total bilirubin (5–21 UI/l)	NA	NA	NA	NA	4	4	NA	NA	4	3	NA	NA	7	NA	7

**Figure 1.**
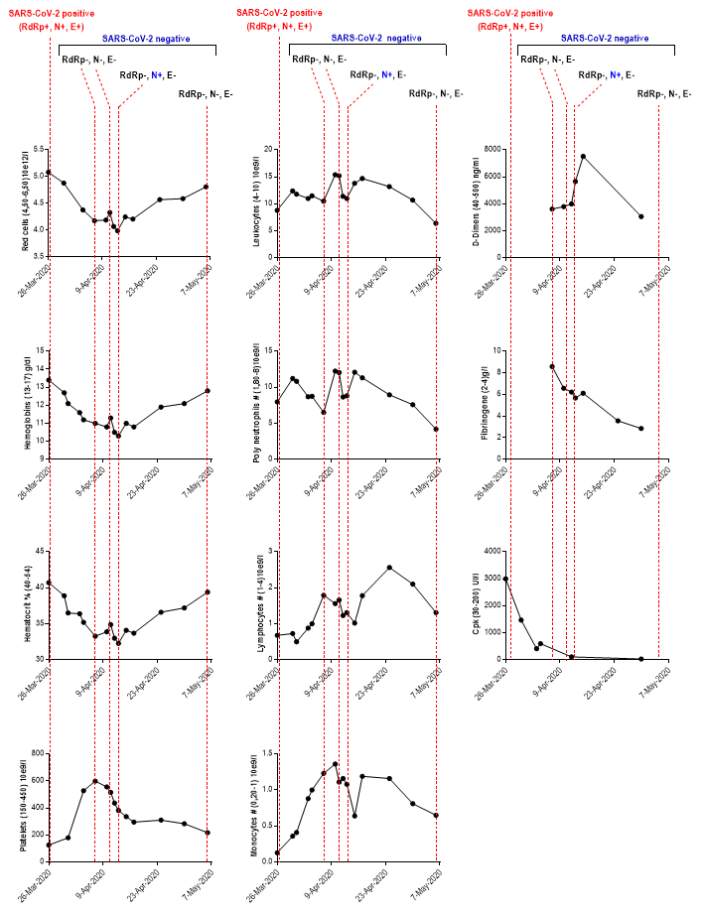
Dynamic profile of laboratory findings in patient with coinfection including SARS-CoV-2 and influenza H1N1. RdRp: RNA-dependent RNA polymerases; N: envelope protein N; S: Spike protein; CpK: creatine phosphokinase; SARS-CoV-2: severe acute respiratory syndrome coronavirus-2; Mar: March; Apr: April; #: Total count. The values between parentheses ‘()’ in the ordinate axis correspond to the reference range values.

Other parameters showed decreased values such as red cells, hemoglobins, hematocrits and mean corpuscular hemoglobin content ( Table 1). The number of leukocytes and neutrophils underwent fluctuations with high rates between April 7 ^th^ and 10 ^th^ ( Figure 1). In this period, bacteriological examination culture revealed infection by additional pathogens, with the presence of yeasts ( *Candidate albicans*) and bacteria ( *Klebsiella pneumoniae*) in bronchial sampling, probably with nosocomial origin. Indeed, *Klebsiella pneumoniae* was isolated on the deep bronchial sample of March 28 ^th^, 2020, five days after his admission and mechanical ventilation. On the 14 ^th^ day of admission (April 4 ^th^ 2020), *Klebsiella pneumoniae* was found once again at 10 ^6^ in a bronchoalveolar lavage which means the failure of treatment with imipenem, meropenem is then introduced. Also, *Candidas albicans* was found in the same bronchoalveolar lavage at 10 ^4^.

The patient stayed at ICU for 26 days from March 24 ^th^ to April 19 ^th^ with orotracheal intubation and using Etomidate (40 mg intravenous dose) for sedation and 120mg of Celocurine for curarization. Enoxaparin (40mg /day) was administered by subcutaneous injection as a preventive anticoagulation up to April 1 ^st^ and increased to 80 mg/day according to the patient being overweight (Body Mass Index 33.8) and to evolution of biological criteria (D-Dimers 1890ng/l, Fibrinogen 10.82g/l, platelets 528x10e9/l). Unfractionated heparin was used as relay according to the high probability of pulmonary embolism. Mechanical ventilation was used with several sessions of prone position and then oxygen therapy on April 19 ^th^. He was treated with hydroxychloroquine for 10 days (Plaquenil, 200mg every eight hours), and by Oseltamivir (4 days, oral suspension 6mg/ml: 75mg twice/day) for influenza H1N1 infection. *Klebsiella pneumoniae* infection was treated using Meropenem (intravenous 1 g every four hours) from 6 ^th^ to 15 ^th^ April and *Enterococcus faecalis* infection was treated using Clamoxyl (intravenous 2 g every eight hours). Venous echodoppler performed on April 14 ^th^ found no thrombosis and no pulmonary embolism.

On April 19 ^th^, the patient was transferred to the pulmonology department where he has a good respiratory evolution allowing oxygen weaning on April 23 ^rd^. He was discharged on April 28 ^th^, receiving kinesitherapy treatment, and taking a preventive anticoagulant therapy (Enoxaparine 4000 IU /0.4ml once daily by SC injection) for three weeks. The CT scan control on April 30 ^th^ 2020 revealed reduction of affection to less than 25% with appearance of more or less symmetrical, predominantly peripheral, bilateral pulmonary ground glass areas. The patient was integrated into the post COVID-19 rehabilitation program.

## Discussion

We report a case of coinfection with SARS-CoV-2 and seasonal influenza H1N1 in a French comorbid patient. The prolonged intensive care and detection of SARS-CoV-2 viral RNA on the bronchoalveolar sample for at least three weeks might be explained by the immunosuppression caused by lung polyinfection (viral, bacterial and fungal) and also by his medical history of pulmonary sarcoidosis with a restrictive ventilatory syndrome and obesity. Indeed, this disease was diagnosed in January 2019 and treated with corticosteroid therapy until December 2019, then in February 2020 under corticosteroids and methotrexate due to the relapse. The patient had a handicap of stage 2 exertional dyspnia according to Medical Research Cuncil (MRC). The patient presented obesity as an additional comorbidity factor.

Bacterial coinfections in COVID-19 patients have been reported in several studies with a rate varying from less than 4% to 8% of bacterial/fungal co-infection cases with some bacterial infections frequently occurred in patients with prolonged hospitalization ^11, 12^. Meanwhile, clinicians should be vigilant for nosocomial bacterial infections ^12^. Different studies have reported co-infection with SARS-CoV-2 and influenza viruses (A or B) ^1– 9^. According to a systematic review and meta-analysis of 3070 COVID-19 patients and 79 patients with concurrent COVID-19 and Influenza infections, the frequency of influenza virus co-infection among patients with COVID-19 was 4.5% and the prevalence of influenza infection was 0.8% in patients confirmed with COVID-19 ^10^. The overall of COVID-19 patients who had influenza co-infection was 1.2%. One other meta-analysis study revealed that co-infection with SARS-CoV-2 and influenza showed a high heterogeneity for overall mortality ^13^. However, the relationship with the severity of COVID19 disease in the case of co-infection associated with one or more comorbidity factors such as diabetes, cardiovascular diseases, hypertension, malignancies, chronic obstructive pulmonary disease, and other comorbidities has been largely reported and could induce a life-threatening situation ^14– 16^. For example, the risk for ICU admissions in COVID-19 individuals with diabetic comorbidity is 14.2% higher than individuals without diabetes ^15^. Cuadrado-Payán *et al.* ^5^ reported that all COVID-19 patients studied with medical history of hypertension, end-stage kidney disease, or type 2 diabetes attended the emergency unit *^5^*. 

The co-detection of SARS-CoV-2 and Influenza H1N1 in our case with comorbidity factor the pulmonary sarcoidosis and obesity demonstrates the challenge to screen in the onset of the respiratory illness for a panel of viruses, which have overlapping clinical patterns and might exacerbate clinical symptoms, increase morbidity and prolong ICU stay. Hence, this case highlights the higher risk and poor outcomes caused by co-infection and the importance to achieve a differential diagnosis of respiratory distress syndromes speciallly in comorbid patients, to recommend seasonal influenza vaccination in addition of SARS-CoV-2 vaccine. It’s necessary to adopt the vigilant preventive measure and therapeutic strategies to prevent a deleterious impact and health service demand on infected and comorbid individuals.

## Consent

Written informed consent was obtained from the patient for the publication of this article and any associated images.

## Data availability

All data underlying the results are available as part of the article and no additional source data are required.
