# Supplementary material for: Case Report: Co-infection with SARS-CoV-2 and influenza H1N1 in a patient with acute respiratory distress syndrome and a pulmonary sarcoidosis
Source: F1000Res. 2022 May 3;9:1482. Originally published 2020 Dec 18. [Version 2] doi: 10.12688/f1000research.26924.2 (PMC9065929; doi:10.12688/f1000research.26924.2)
Supplement: Peer review history

# Peer review: Reviewer response for version 2

**DOI:** 10.5256/f1000research.129014.r136555

**Published:** 2022-05-03

**Authors:** Louise E. Lansbury

**Affiliations:** 1 Division of Epidemiology and Public Health, University of Nottingham, Nottingham, UK; 2 NIHR Nottingham Biomedical Research Centre, Nottingham, UK

Thank you for your satisfactory responses to my comments. I have no further comments to add.

Are enough details provided of any physical examination and diagnostic tests, treatment given and outcomes?

Yes

Is the case presented with sufficient detail to be useful for other practitioners?

Partly

Is sufficient discussion included of the importance of the findings and their relevance to future understanding of disease processes, diagnosis or treatment?

Partly

Is the background of the case’s history and progression described in sufficient detail?

Yes

Reviewer Expertise:

Respiratory pathogens

I confirm that I have read this submission and believe that I have an appropriate level of expertise to confirm that it is of an acceptable scientific standard.

# Peer review: Reviewer response for version 1

**DOI:** 10.5256/f1000research.29738.r86503

**Published:** 2021-07-20

**Authors:** Khalid Sadki

**Affiliations:** 1 Laboratory of Human Pathologies Biology, Faculty of Sciences, Mohammed V University, Rabat, Morocco

The work submitted by the authors is an interesting case report. They report a French patient co-infected by two severe viruses, SARS-CoV-2 and 2009 H1N1 Influenza strain.

 The most important physical examination and diagnostic tests are reported in the case report. The methodology followed is well reported. However, data concerning the genetic investigations for both viruses is missed. It is recommended to discuss it, taking into consideration the specific phenotype observed in this case.

Are enough details provided of any physical examination and diagnostic tests, treatment given and outcomes?

Yes

Is the case presented with sufficient detail to be useful for other practitioners?

Yes

Is sufficient discussion included of the importance of the findings and their relevance to future understanding of disease processes, diagnosis or treatment?

Partly

Is the background of the case’s history and progression described in sufficient detail?

Yes

Reviewer Expertise:

Immunology and immunogenetics

I confirm that I have read this submission and believe that I have an appropriate level of expertise to confirm that it is of an acceptable scientific standard.

# Response

**Published:** 2022-03-24

**Authors:** lekbir BAALA

**Affiliations:** Centre Hospitalier Régionald'Orléans & CNRS Orléans, France

I am Dr. BAALA, a co-author of this article, I thank Pr. SADKI for his review and I am pleased to respond to his comments:

*We didn't give more details of the molecular data used for the SARS-CoV-2 and H1N1 tests since we were limited by the number of words not to be exceeded in the article. However, we may include some Ct data for SARS-CoV2 to indirectly indicate viral load kinetics.*

*As Pr Sadki and Pr Lansbury (first reviewer) suggested regarding the phenotype of the patient, the fact that the co-infection by SARS-Cov-2 and H1N1 viruses occurred in a patient with a history of pulmonary sarcoidosis, we will give more notice to the role of this immunosuppression history and poor outcome in the discussion.*

# Peer review: Reviewer response for version 1

**DOI:** 10.5256/f1000research.29738.r86504

**Published:** 2021-06-21

**Authors:** Louise E. Lansbury

**Affiliations:** 1 Division of Epidemiology and Public Health, University of Nottingham, Nottingham, UK; 2 NIHR Nottingham Biomedical Research Centre, Nottingham, UK

In this case report, Baala *et al.* describe the clinical course of a patient with a history of pulmonary sarcoidosis who was co-infected with SARS-CoV-2 and influenza A (H1N1) virus and developed acute respiratory distress syndrome, requiring a prolonged ICU stay. The case is generally well-described and a useful reminder that the clinical presentation of SARS-CoV2 and influenza infections may overlap, and of the importance of the timely recognition of co-infection of SARS-CoV2 with other respiratory pathogens. However, the authors may wish to consider the following points:

**Abstract:**

- Please clarify the sentence: “Co-infection of SARS-CoV-2 and other respiratory syndrome has rarely been reported”. Do you mean: "Co-infection with SARS-CoV2 and other respiratory **pathogens**"?
- Italicisation of ‘respiratory distress syndrome’ is not required (also italicised in the title

**Introduction:**

- “To date, there is no published case with a co-infection between SARS-CoV-2 and influenza H1N1”. This can no longer be claimed as there are now several reports of co-infections with these viruses from several countries.

**Case Report:**

- “The SARS-CoV-2 RT-PCR test was positive on March 22 ^nd^ 2020.” Is there information on the date of onset of symptoms in relation to the timing of this test?
- Did the patient receive the seasonal influenza vaccine prior to the 2019-2020 influenza season?
- Would it be possible to present the chest CT-scan?
- How many days had the patient been in ICU before *Candida albicans* and *Klebsiella pneumoniae* were first isolated?
- The authors indicate that an infection with *Enterococcus faecalis* infection was also treated; what was believed to be the source of this infection?

**Discussion:**

- As per the previous comment, this is no longer the first case report of a co-infection with SARS-CoV-2 and influenza A (H1N1). However, the fact that it occurred in a patient with a history of pulmonary sarcoidosis is perhaps more original and may be worth emphasising more in the discussion (and perhaps also in the title?).
- The effect of SARS-CoV-2 and influenza co-infection on prognosis could perhaps be considered in a little more detail in the Discussion. The studies cited (Ding *et al.,* Cuadrado-Payán *et al.*) indicated that the clinical and analytic courses of their patients did not differ from patients infected only with SARS-CoV-2. However, other studies have suggested that co-infection may be associated with worse outcomes (e.g. see Stowe *et al*. ^1^ and Hashemi *et al.* ^2^).

Overall, I believe the case study contributes to the literature on patients who are co-infected with SARS-CoV-2 and influenza as there are many uncertainties regarding these patients. I support indexing but would recommend the clarifications I have suggested are considered.

Are enough details provided of any physical examination and diagnostic tests, treatment given and outcomes?

Yes

Is the case presented with sufficient detail to be useful for other practitioners?

Partly

Is sufficient discussion included of the importance of the findings and their relevance to future understanding of disease processes, diagnosis or treatment?

Partly

Is the background of the case’s history and progression described in sufficient detail?

Yes

Reviewer Expertise:

Respiratory pathogens

I confirm that I have read this submission and believe that I have an appropriate level of expertise to confirm that it is of an acceptable scientific standard, however I have significant reservations, as outlined above.

## References

[1] : Interactions between SARS-CoV-2 and influenza, and the impact of coinfection on disease severity: a test-negative design. *Int J Epidemiol* .2021; 10.1093/ije/dyab081 10.1093/ije/dyab081 33942104PMC8135706

[2] : Co‐infection with COVID‐19 andinfluenza A virus in two died patients with acute respiratory syndrome, Bojnurd, Iran. *Journal of Medical Virology* .2020;92(11) : 10.1002/jmv.26014 2319-2321 10.1002/jmv.26014 PMC727290832410338

# Response

**Published:** 2022-03-18

**Authors:** lekbir BAALA

**Affiliations:** Centre Hospitalier Régionald'Orléans & CNRS Orléans, France

Dear All,

 I'm Dr. Lekbir BAALA, the author of this article. I thank Dr. Louise E. Lansbury for her comments and I'm pleased to give answers below in italics.

**Abstract:**

**• Please clarify the sentence: “Co-infection of SARS-CoV-2 and other respiratory syndrome has rarely been reported”. Do you mean: "Co-infection with SARS-CoV2 and other respiratory pathogens"?**

- *Yes, we mean* *co-infection with SARS-CoV2 and other respiratory pathogens.* *it’s corrected.*

• **Italicisation of ‘respiratory distress syndrome’ is not required (also italicised in the title**

- *Ok, it was corrected.*

**Introduction:**

**• “To date, there is no published case with a co-infection between SARS-CoV-2 and influenza H1N1”. This can no longer be claimed as there are now several reports of co-infections with these viruses from several countries**.

- *Yes, as we submitted our case report in December 2020, at that time no case with H1N1 and SARS-CoV-2 co-infection has been published in PUBMED, but now it is. We have changed the sentence.*

**Case Report:**

**• “The SARS-CoV-2 RT-PCR test was positive on March 22nd 2020.” Is there information on the date of onset of symptoms in relation to the timing of this test**?

- *Yes, the PCR was positive on 22nd March 2020.* *The cough and fever had already been evolving for three to four days, which is what motivated screening (clinical investigation).*

**• Did the patient receive the seasonal influenza vaccine prior to the 2019-2020 influenza season?**

- *Not known as vaccinated against influenza.*

• **Would it be possible to present the chest CT-scan?**

- *We have obtained the chest CT Scan for the patient.* *The conclusion was:* “Appearance of more or less symmetrical, predominantly peripheral, bilateral pulmonary ground glass areas compatible with Covid 19 type pneumopathy in the context of the epidemic...”.

**• How many days had the patient been in ICU before Candida albicans and Klebsiella pneumoniae were first isolated?**

- *Klebsiella pneumonia was isolated on the deep bronchial sample of March 28, 2020, so five days after his admission and mechanical ventilation, he was on C3G (ceftriaxone) empirically at that time.*
- *On the 14th day of admission (06/04/2020), Klebsiella pneumonia was found once again at 10^6 in a bronchoalveolar lavage which means the failure of treatment with imipenem, meropenem is then introduced. Also found in this bronchoalveolar lavage candidas albicans 10* *^4.*

* *
**• The authors indicate that an infection with Enterococcus faecalis infection was also treated; what was believed to be the source of this infection?**

- *It is probably the digestive tract, the patient had presented abdominal pain, diarrhea, and vomiting which were attributed to poor tolerance of enteral nutrition. There was no specific digestive exploration because the evolution of this disorder was rapidly favorable.*

**Discussion:**

**• As per the previous comment, this is no longer the first case report of a co-infection with SARS-CoV-2 and influenza A (H1N1). However, the fact that it occurred in a patient with a history of pulmonary sarcoidosis is perhaps more original and may be worth emphasising more in the discussion (and perhaps also in the title?).**

- **We take into account this relevant remark on the prevalence* *. One* *meta-analysis (June 2021) of prevalence studies revealed that the frequency of influenza virus (A/B) co-infection among patients with COVID-19 was 4.5% in Asia, and 0.4 % from the American continent. This meta-analysis indicated that overall 1.2% of COVID-19 patients had influenza co-infection.**
- ** *
*We will add to the discussion the notion of the vulnerability of the immunocompromised (in our case sarcoidosis under methotrexate)**

**• The effect of SARS-CoV-2 and influenza co-infection on prognosis could perhaps be considered in a little more detail in the Discussion. The studies cited (Ding et al., Cuadrado-Payán et al.) indicated that the clinical and analytic courses of their patients did not differ from patients infected only with SARS-CoV-2. However, other studies have suggested that co-infection may be associated with worse outcomes (e.g. see Stowe et al.1 and Hashemi et al.2).**

- *Indeed the data in the literature are divergent, but a large series of patients are needed or a review of the literature is probably necessary to conclude. The recent review *meta-analyses (June 2021) indicated that* *the outcomes of COVID-19-Influenza co-infection were reported in 11 studies with 2 of 29 patients who died (6.9%), and 17 out of 29 patients recovered (58.6%), but more studies are needed to evaluate the exact* *effect of SARS-CoV-2 and influenza co-infection on prognosis* *and clinical outcomes (Masoud Dadashi et al. 2021).**
- **The impact of co-infection with SARS-CoV-2 and influenza (H1N1) have been recently developed in animal models. For example, the sequential infection with H1N1 and SARS-CoV-2 aggravated COVID-19 pathogenesis in a mammalian model (Linlin Bao Signal Transduct Target Ther 2021). They conclude that* *co-infection with H1N1 and SARS-CoV-2 extended the duration of clinical manifestation of COVID-19, and enhanced pulmonary damage.**
